# RETRACTED: Tishkevich et al. Magnetic Properties of the Densely Packed Ultra-Long Ni Nanowires Encapsulated in Alumina Membrane. *Nanomaterials* 2021, *11*, 1775

**DOI:** 10.3390/nano16191262

**Published:** 2026-10-07

**Authors:** Daria Tishkevich, Alla Vorobjova, Dmitry Shimanovich, Egor Kaniukov, Artem Kozlovskiy, Maxim Zdorovets, Denis Vinnik, Andrei Turutin, Ilya Kubasov, Alexander Kislyuk, Mengge Dong, M. I. Sayyed, Tatiana Zubar, Alex Trukhanov

**Affiliations:** 1 Laboratory of Magnetic Films Physics, Scientific-Practical Materials Research Centre of National Academy of Sciences of Belarus, 220072 Minsk, Belarus; fix.tatyana@gmail.com; 2 Laboratory of Single Crystal Growth, South Ural State University, 454080 Chelyabinsk, Russia; denisvinnik@gmail.com; 3 Department of Micro and Nanoelectronics, Belarusian State University of Informatics and Radioelectronics, 220013 Minsk, Belarus; vorobjova@bsuir.by (A.V.); shdl@tut.by (D.S.); 4 Department of Technology of Electronic Materials, Department of Materials Science of Semiconductors and Dielectrics, National University of Science and Technology, «MISIS», 119049 Moscow, Russia; ka.egor@mail.ru (E.K.); aturutin92@gmail.com (A.T.); kubasov.ilya@gmail.com (I.K.); akislyuk94@gmail.com (A.K.); 5 Engineering Profile Laboratory, L.N. Gumilyov Eurasian National University, Nur-Sultan 010000, Kazakhstan; artem88sddt@mail.ru (A.K.); mzdorovets@inp.kz (M.Z.); 6 Laboratory of Solid State Physics, Institute of Nuclear Physics, Almaty 050032, Kazakhstan; 7 Department of Intelligent Information Technologies, Ural Federal University Named after the First President of Russia B.N. Yeltsin, 620075 Yekaterinburg, Russia; 8 Department of Physics and I3N, University of Aveiro, 3810-193 Aveiro, Portugal; 9 Department of Resource and Environment, Northeastern University, Shenyang 110819, China; mg_dong@163.com; 10 Department of Physics, Faculty of Science, Isra University, Amman 11622, Jordan; dr.mabualssayed@gmail.com; 11 Department of Nuclear Medicine Research, Institute for Research and Medical Consultations (IRMC), Imam Abdulrahman bin Faisal University (IAU), Dammam 31441, Saudi Arabia

The journal retracts the article “Magnetic Properties of the Densely Packed Ultra-Long Ni Nanowires Encapsulated in Alumina Membrane” [1] cited above.

Following publication, concerns were brought to the attention of the Editorial Office regarding the integrity of an image within this article.

In accordance with standard journal procedures, the Editorial Office and Editorial Board conducted an investigation that identified indications of inappropriate image editing within Figure 5 presented within this publication. As the data presented in Figure 5 support key findings of this study, and in the absence of a response from the authors to address the identified concerns, the Editorial Board no longer has confidence in the reliability of the article and has therefore decided to retract it in accordance with MDPI’s retraction policy.

This retraction was approved by the Editor-in-Chief of *Nanomaterials*.

The authors have been informed of this retraction but did not provide a comment on this decision.
